# Memory T Cells Discrepancies in COVID-19 Patients

**DOI:** 10.3390/microorganisms11112737

**Published:** 2023-11-09

**Authors:** Hajir A. Al Saihati, Hosni A. M. Hussein, Ali A. Thabet, Ahmed A. Wardany, Sabry Y. Mahmoud, Eman S. Farrag, Taha I. A. Mohamed, Samah M. Fathy, Mohamed E. Elnosary, Ali Sobhy, Abdelazeem E. Ahmed, Ahmed M. El-Adly, Fareed S. El-Shenawy, Asmaa A. Elsadek, Amal Rayan, Zeinab Albadry M. Zahran, Omnia El-Badawy, Mohamed G. M. El-Naggar, Magdy M. Afifi, Asmaa M. Zahran

**Affiliations:** 1 Department of Clinical Laboratory Sciences, College of Applied Medical Science, University of Hafr Al Batin, P.O. Box 1803, Hafar Al Batin 31991, Saudi Arabia; hajirsh@uhb.edu.sa (H.A.A.S.); esfarrag@uhb.edu.sa (E.S.F.); 2 Department of Botany and Microbiology, Faculty of Science, Al-Azhar University, Assiut 71524, Egypt; ahmed_wr2000@azhar.edu.eg (A.A.W.); taha_mohamed@yahoo.com (T.I.A.M.); ahmedeladly.ast@azhar.edu.eg (A.M.E.-A.); fareedsh73@yahoo.com (F.S.E.-S.); 3 Department of Zoology, Faculty of Science, Al-Azhar University, Assiut 71524, Egypt; athabet@azhar.edu.eg; 4 Biology Department, College of Sciences, University of Hafr Al-Batin, Hafr Al-Batin 31991, Saudi Arabia; symohamed@uhb.edu.sa; 5 Department of Microbiology, Sohag University, Sohag 82524, Egypt; 6 Department of Microbiology, South Valley University, Qena 83523, Egypt; 7 Department of Zoology, Faculty of Science, Fayoum University, Fayoum 63514, Egypt; samahfathy1972@gmail.com; 8 Department of Botany and Microbiology, Faculty of Science, Al-Azhar University, Nasr City 11884, Egypt; mohamed.elnosary@azhar.edu.eg (M.E.E.); afifi_magdy@ymail.com (M.M.A.); 9 Department of Clinical Pathology, Faculty of Medicine, Al-Azhar University, Assiut 71524, Egypt; dralisobhy@azhar.edu.eg (A.S.); abd_elazeem81@yahoo.com (A.E.A.); 10 Department of Virology, Chest Hospital, Assiut 71514, Egypt; amy62014@gmail.com; 11 Department of Clinical Oncology, Faculty of Medicine, Assiut University, Assiut 71515, Egypt; amalrayan@aun.edu.eg; 12 Department of Clinical Pathology, Faculty of Medicine, Assiut University, Assiut 71515, Egypt; zeinab_albadry@aun.edu.eg; 13 Department of Medical Microbiology and Immunology, Faculty of Medicine, Assiut University, Assiut 71515, Egypt; omniaalbadawy@aun.edu.eg; 14 Department of Clinical Pathology, South Egypt Cancer Institute, Assiut University, Assiut 71515, Egypt; mohamed1979@aun.edu.eg (M.G.M.E.-N.); asmaa.zahran@yahoo.com (A.M.Z.)

**Keywords:** COVID-19, SARS-CoV-2, T cell subtypes, memory T cells

## Abstract

The immune response implicated in Coronavirus disease 2019 (COVID-19) pathogenesis remains to be fully understood. The present study aimed to clarify the alterations in CD4^+^ and CD8^+^ memory T cells’ compartments in SARS-CoV-2-infected patients, with an emphasis on various comorbidities affecting COVID-19 patients. Peripheral blood samples were collected from 35 COVID-19 patients, 16 recovered individuals, and 25 healthy controls, and analyzed using flow cytometry. Significant alterations were detected in the percentage of CD8^+^ T cells and effector memory-expressing CD45RA CD8^+^ T cells (TEMRA) in COVID-19 patients compared to healthy controls. Interestingly, altered percentages of CD4^+^ T cells, CD8^+^ T cells, T effector (TEff), T naïve cells (TNs), T central memory (TCM), T effector memory (TEM), T stem cell memory (TSCM), and TEMRA T cells were significantly associated with the disease severity. Male patients had more CD8^+^ TSCMs and CD4^+^ TNs cells, while female patients had a significantly higher percentage of effector CD8^+^CD45RA^+^ T cells. Moreover, altered percentages of CD8^+^ TNs and memory CD8^+^CD45RO^+^ T cells were detected in diabetic and non-diabetic COVID-19 patients, respectively. In summary, this study identified alterations in memory T cells among COVID-19 patients, revealing a sex bias in the percentage of memory T cells. Moreover, COVID-19 severity and comorbidities have been linked to specific subsets of T memory cells which could be used as therapeutic, diagnostic, and protective targets for severe COVID-19.

## 1. Introduction

Coronavirus disease 2019 (COVID-19), caused by the recently identified severe acute respiratory syndrome coronavirus 2 (SARS-CoV-2), is a serious disease that has resulted in widespread global morbidity and mortality [1,2]. SARS-CoV-2 is a member of the coronavirus (CoVs) family that is characterized by its single-stranded, and positive-sense RNA genome [3]. CoVs infect many mammals and cause a wide variety of diseases including respiratory and neurological diseases [4]. In response to SARS-CoV-2 infection, individuals develop viral-specific CD4^+^ cells and CD8^+^ memory T cells as well as antibodies [5,6]. Several studies of acute and convalescent COVID-19 patients have revealed that T cell responses are associated with reduced severity of the disease, implying that CD4^+^ T and CD8^+^ T cell responses are important for the resolution and the control of infection [7,8,9]. Moreover, dysregulation in innate and adaptive immune responses is critical for infection outcome and disease progression [10]. Immune memory from primary infection or immunization has been shown to provide protection against subsequent infections [11,12]. Consequently, vaccination against SARS-CoV-2 depends mainly on the immunologic memory, which includes CD4^+^ and CD8^+^ T cells, as well as their effector and memory subtypes.

CD4^+^ T cells play a critical role in both innate and adaptive immunity [13,14]. During the primary immune response, CD4^+^ T cells are activated in the secondary lymphoid organs, where they augment the anti-pathogen response by driving B-cell germinal responses and supporting CD8^+^ T-cell activation [14]. Activated CD4^+^ T cells then migrate from secondary lymphoid organs to the infected sites, where they participate in controlling the infection [14]. It is noteworthy that, most CD4^+^ T cells undergo apoptosis, while the remaining 10% form long-lived memory cells that are responsible for the immune response upon secondary infection [14]. These memory cells retain the characteristics of the initial CD4^+^ T cells they are derived from and are consequently divided based on their functional responses [15,16].

Naïve CD4^+^ T cells, developed after primary infection, differentiate into many types of memory T cells that preserve information about previous infection [14,17]. Upon antigen presentation to naive T cells, they are stimulated, proliferated, and transformed into effector cells that move to the pathogen site to eliminate it [14,17]. It is well known that the effector cells have a short life span, whereas the subsets of memory cells possess a long-term survival potential [14,17]. These memory cells include many subsets. The non-circulating memory cells continue to reside in the sites of infection, forming tissue resident memory T cells (TRMs). The other subset is made of circulating memory cells, including the central memory cells (TCMs), situated at secondary lymphoid organs, and the effector memory cells (TEMs) that can be found in the newly infected tissues. [14,17]. A special long-lived memory T-cell subtype that is believed to be made of naïve-like T cell derivatives is known as stem cell memory T cells (TSCM). This subset of memory T cells demonstrates stem cell-like characteristics with their capacity to self-renew and to generate a more differentiated progeny following infection and antigen stimulation [17]. Interestingly, TSCM cells represent 2 to 4% of both CD4^+^ and CD8^+^ T cell populations [17,18].

Memory CD8^+^ T cells are antigen-specific and long-lived population of T cells that give enhanced immune response upon encountering with the same antigen [19]. Unique subsets of CD8^+^ T memory cell populations have been identified through the expression of different sets of cell surface markers [20]. Due to the presence of few numbers of naïve CD8^+^ T cells in draining lymph nodes that respond to pathogens, the time required for generating the primary response makes the patient vulnerable to deleterious damages [19]. Consequently, resident memory CD8^+^ T cells that can respond efficiently and rapidly to these infections are critical and valuable [19]. Some pathogens directly attack lymphatic vessels and draining lymph nodes. These pathogens can be efficiently eliminated by the circulating memory cells, either TEMs or TCMs [21]. Compared to TEMs, TCMs are characterized by higher capacity for robust proliferation upon reactivation [22]. In humans, TCMs have been phenotypically identified as CD45RA^−^ CD45RO^+^ CCR7^+^ CD62L^+^ cells and primarily reside in lymphoid tissues such as lymph nodes. On the other hand, TEM cells migrate from lymphoid tissue to peripheral tissues and are characterized by lacking the expression of CCR7 and CD62L phenotypic markers. TEMs are poised for rapid effector function, making them more immediately responsive to reinfections [23]. TCM and TEM cells respond differently following T cell receptors’ (TCRs) triggering and activation. It has been shown that, upon activation of TCRs, TEM cells respond immediately and express a high level of perforin and INF-γ, while TCM cells mostly release huge amount of IL-2 and can differentiate into both T effector (TEff) and TEM cells [23].

Previous studies have suggested a correlation between immune response against SARS-CoV-2 and disease severity [15]. It is noteworthy that, it has been reported that memory CD4^+^ T cells facilitate protective immunity towards CoV-strains such as SARS-CoV [9] and MERS [17], whereas CD8^+^ memory T cell responses have been recorded in SARS-CoV convalescent patients [18]. Nevertheless, there are conflicting results reported regarding the percentages of T memory cells in the context of SARS-CoV-2 infection. For example, one report provided evidence for a decline in both T memory and T regulatory cells in severely infected COVID-19 patients [19]. This finding has been explained by the exaggerated inflammatory response resulting from the immune disturbance. On the contrary, a clear distinction was observed between memory T cells obtained from individuals with acute severe or acute non-severe COVID-19 and those derived from convalescent and healthy control subjects [21]. Another study observed strong SARS-CoV-2-specific CD4^+^ and CD8^+^ T cell responses in the majority of convalescent patients, with significantly larger overall T cell responses in those who had severe symptoms compared with mild disease. However, there was a greater proportion of CD8^+^ T cell compared with CD4^+^ T cell responses in the mild cases [22].

Drawing on insights gleaned from prior research, it becomes evident that discrepancies in the percentage and behavior of CD4^+^ or CD8^+^ T cells are characteristic of COVID-19. However, the intricacies of these observations remain incompletely understood, necessitating further comprehensive investigations to clarify thoroughly the role of SARS-CoV-2 specific memory T cell response. Moreover, the correlation between different subsets of memory T cells and COVID-19 severity, and its associated comorbidities, needs further elucidation. Consequently, the current study has been designed to investigate the differences in the memory T cell response among SARS-CoV-2 infected patients compared to healthy controls through assessing the proportions of several subsets of CD4^+^ and CD8^+^ memory T cells, taking into consideration different comorbidities affecting COVID-19 patients.

## 2. Material and Methods

### 2.1. Patients and Subjects

Thirty-five COVID-19 patients with a confirmed SARS-CoV-2 positive result were recruited for participation together with sixteen recovered COVID-19 and twenty-five healthy controls. The diagnosis of COVID-19 was based on Real-Time polymerase chain reaction (RT-PCR) on throat swabs for all patients and controls. COVID-19 patients and health controls were subjected to detailed history, clinical examinations, and RT-PCR. Healthy control individuals were normal without history of chronic diseases or close contact with COVID-19 patients within the preceding two weeks of sample collection, and with normal hematologic parameters. Peripheral blood samples were collected from COVID-19 patients, recovered, and healthy controls for flow cytometric detection of immune memory T cells and evaluation of complete blood picture (CBC), D-dimer, C-reactive protein, and ferritin evaluation. We analyzed CBC and C reactive protein using Ruby Cell Dyn (American, Serial number: 36026BG) and Mispa Automated specific protein analyzer (AGAPPE, Cham, Switzerland), respectively. Ferritin and D-dimer were measured using the mini-VIDAS^®^ system (Biomerieux SA, Marcy-l’Étoile, France).

COVID-19 patients were categorized into severe and non-severe cases based on the Egyptian Ministry of Health (MOH) and World Health Organization guidelines [24,25,26].

The present study was approved by the ethical committees of Al-Azhar University in Assiut, Egypt (APPROVAL NUMBER/ID: 6/2021) and Hafr Al Batin University in Hafar Al Batin, Saudi Arabia (APPROVAL NUMBER/ID: HAPO-05-FT-119). Written informed consents were signed by all participants or their surrogates after describing the study purposes and procedures.

### 2.2. Flow Cytometric Detection of Subset of T Lymphocytes

**Figure 1:**
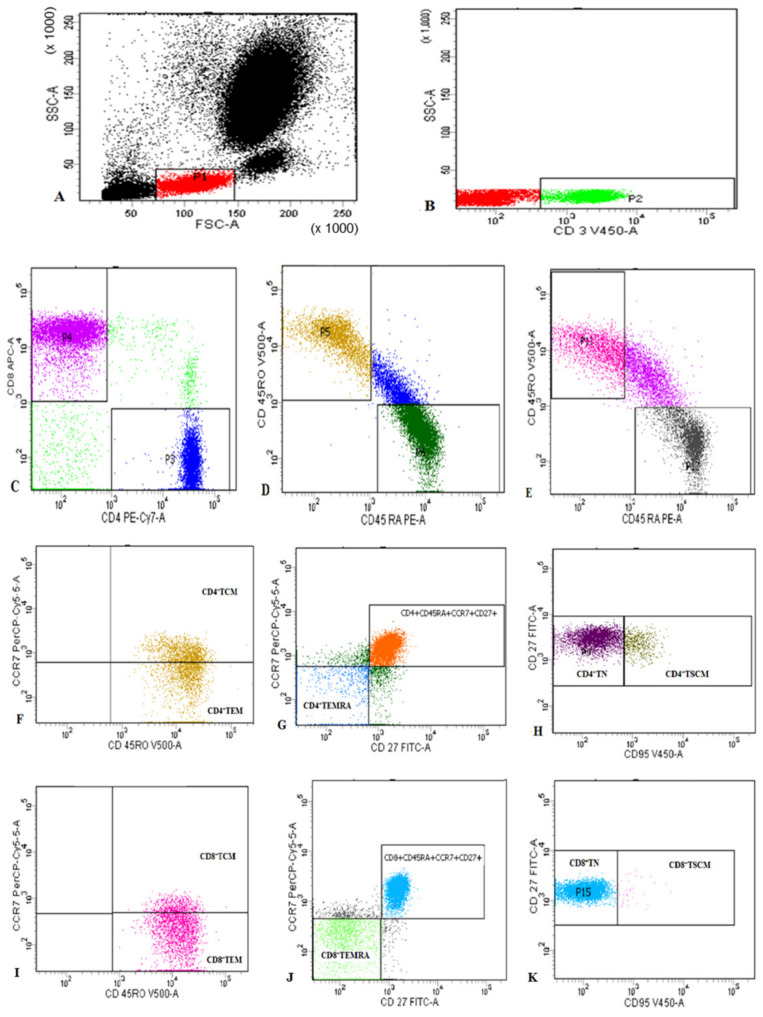
Representative flow cytometric detection of T lymphocyte subsets: (**A**): Lymphocytes were gated based on their characteristics on forward and side scatter histogram. (**B**): Then CD3^+^ T cells were assessed on lymphocytes and then gated for further analysis of CD4 and CD8. (**C**): CD4^+^ cells and CD8^+^ T cells were assessed on CD3^+^ lymphocytes and then gated for further analysis. (**D**–**K**): CD4^+^ cells and CD8^+^ T cells were subdivided based on characteristic expression patterns of CD45RA, CD45RO, CD27, CCR7, and CD95 into: (**F**): CD4^+^TCM (CD4^+^CD45RO^+^CCR7^+^) and CD4^+^TEM (CD4^+^CD45RO^+^CCR7^−^), (**G**) CD4^+^TEMRA (CD4+CD45RO^−^CD45RA^+^CCR7^−^CD27^−^), (**H**) CD4^+^TN (CD4^+^CD45RO^−^CD45RA^+^CCR7^+^CD27^+^CD95^−^) and CD4^+^TSCM; CD4^+^CD45RO^−^CD45RA^+^CCR7^+^CD27^+^CD95^+^), (**I**): CD8^+^TCM (CD8^+^CD45RO^+^CCR7^+^) and CD8^+^TEM (CCD8^+^CD45RO^+^CCR7^−^), (**J**) CD8^+^TEMRA (CD8^+^CD45RO^−^CD45RA^+^CCR7^−^CD27^−^), and (**K**) CD8^+^TN (CD8^+^CD45RO^−^CD45RA^+^CCR7^+^CD27^+^CD95^−^) and CD8^+^TSCM (CD8^+^CD45RO^−^CD45RA^+^CCR7^+^CD27^+^CD95^+^.

Freshly collected peripheral blood samples were stained for flow cytometric analysis as previously described [27]. Samples were stained for 20 min at 4 °C with V500-congugated anti-CD45RO, Fluorescein isothiocyanate (FITC)-conjugated anti-CD27, allophycocyanin (APC)-H7-conjugated anti-CD3, phycoerythrin-cyanine 7 (PE-CY7)-conjugated anti-CD4, APC-conjugated anti-CD8, peridinin-chlorophyll-protein (PerCP)-conjugated anti-CCR7, PE-conjugated anti-CD45RA, and V450-conjugated CD95 (Becton Dickinson Biosciences, San Jose, CA, USA). Then, 10 µL of each of the monoclonal antibodies were added to 100 µL of the sample. Following incubation, RBCs lysis using Lysing Buffer (catalogue number: 555899, BD Pharm Lyse™, Becton Dickinson Biosciences, San Jose, CA, USA) and washing were performed. Then the cells were resuspended in PBS. An isotype-matched negative control anti-human IgG was used with each sample. Flow cytometric analysis was carried out using a BD FACSCanto II analyzer equipped with three lasers (Becton Dickinson Biosciences, San Jose, CA, USA). The instrument was set up using BD Cytometer Setup and Tracking (CS&T) beads. BD FACSDiva software (v6.1.3) was used for data acquisition and analysis. Application settings were established to optimize the cytometer’s photomultiplier tube (PMT) voltages [28]. Lymphocytes were assessed based on their forward and side scatter characteristics. Then, CD3^+^ T cells were detected within the lymphocytes and gated for further analysis of CD4 and CD8. CD4^+^ and CD8^+^ T cells were then gated. Assessment of CD45RA and CD45RO on both CD4^+^ and CD8^+^ T cells, followed by their gating for further analysis of CD27, CCR7, and CD95 expression was operated. Each T cell subset was defined as shown in Figure 1.

### 2.3. Statistics

Regarding the immune cells, Shapiro–Wilk test explored that CD3^+^ T (*p* = 0.3), CD8^+^ T (*p* = 0.2), CD4^+^CD45^+^RO (*p* = 0.3), and CD4^+^ TCM (*p* = 0.1) were normally distributed while the remaining immune cells were not normally distributed with *p* < 0.05. All data were analyzed using IBM-SPSS ver. 26; for comparisons between two categorical variables, the Mann–Whitney test and independent sample *t*-test were used, for the ordinal or nominal variables χ^2^ test was used. All results were considered significant at *p*-value ≤ 5%.

## 3. Results

### 3.1. Demographics, Comorbidities, and Laboratory Characteristics of COVID-19 Patients

Demographics of COVID-19 patients are illustrated in Table 1. The median age of recruited patients was 60 years, and 45.7% (16 patients) of them were older than 60. More than 50% of patients were male, hypertensive, and diabetic, while 77.1% of them were considered severe COVID-19 cases. Most of the inflammatory markers, like D-dimer, ferritin, CRP, and other hematologic parameters, were above baseline together with lymphopenia (mean percentage = 9.0 ± 1.0), but had normal other hematologic parameters (Table 2). The distribution of different hematologic parameters was comparable between severe and non-severe cases.

### 3.2. The Changes in the Percentages of CD4^+^ and CD8^+^ T Cells and Their Memory Subsets in Patients, Recovered, and Control Groups

**Figure 2:**
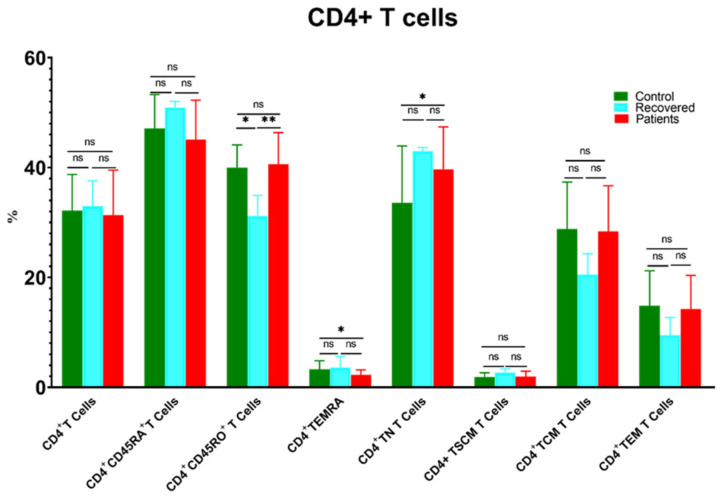
Comparison of the percentages of CD4^+^ T cells and their memory subsets in COVID-19 patients versus recovered and healthy controls. The ns indicated no significant differences among groups and the *p* values: * *p* < 0.05 and ** *p* < 0.01 indicated the significant correlation among different groups.

The level of CD4^+^ T cells showed a comparable distribution between different groups (Figure 2). Unexpectedly, the level of CD4^+^ T naïve cells (TNs) was significantly higher in patients compared to controls. Conversely, CD4^+^ TEMRA cells were accumulated in controls compared to patients. Moreover, CD4^+^CD45RO^+^ T cells decreased substantially in the recovered group relative to the healthy control and patient groups (Figure 2).

**Figure 3:**
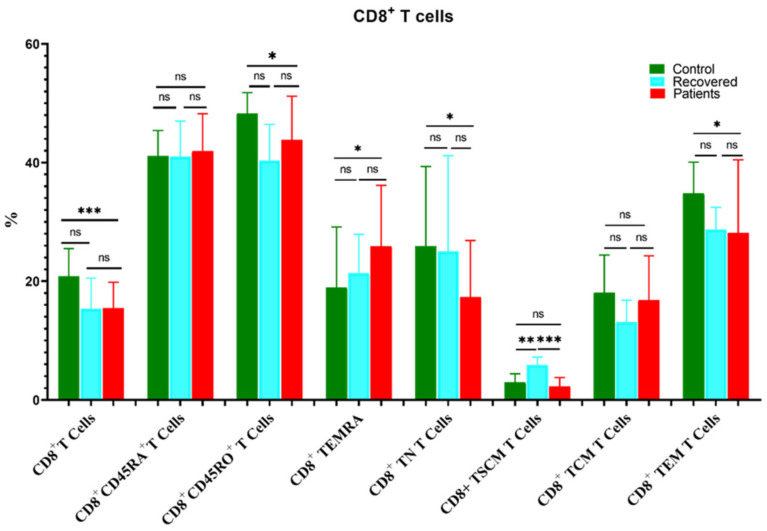
Comparison of the percentages of CD8^+^ T cells and their memory subsets in COVID-19 patients versus recovered and healthy controls. The ns indicated no significant differences among groups and the *p* values: * *p* < 0.05, ** *p* < 0.01 and *** *p* < 0.001 indicated the significant correlation among different groups.

Similarly, the percentage of CD8^+^ T cells decreased significantly in COVID-19 patients compared to the healthy controls group (Figure 3). Moreover, CD8^+^CD45RO^+^, CD8^+^ TNs, and CD8^+^ TEM were substantially reduced in patients compared to healthy controls, whereas CD8^+^ TEMRA cells were higher in patients relative to the control group (Figure 3). Furthermore, CD8^+^ TSCM was higher in the recovered group compared to the patient and healthy control groups.

### 3.3. Differences in the Percentage of Immune Cells According to Sex of COVID-19 Patients

**Figure 4:**
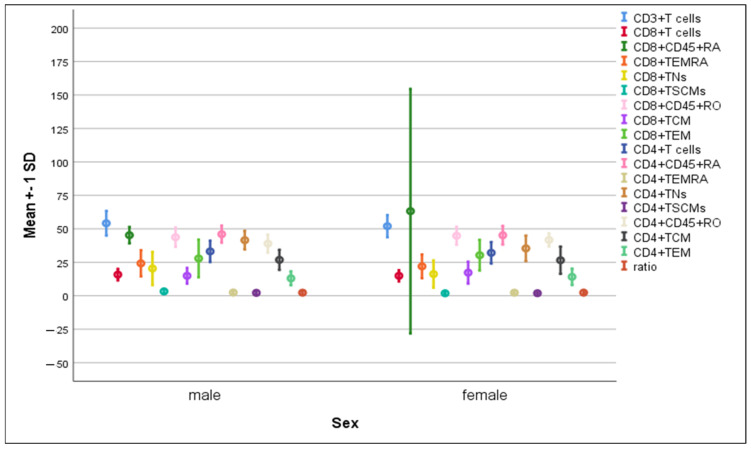
Differences in the percentage of immune cells according to sex. Data are expressed as mean ± SD.

There was a significant rise in the percentages of CD8^+^ TSCMs (3.23 ± 1.9 vs. 1.87 ± 1.0, *p* = 0.05) and CD4^+^ TNs (41.53 ± 7.05 vs. 35.38 ± 9.6, *p* = 0.037) in males compared to females, while the mean percentage of CD8^+^CD45RA^+^ cells was significantly higher in females compared to males (63.2 ± 91.4 vs. 45.26 ± 6.2, *p* = 0.049). Meanwhile, the remaining immune cells exhibited comparable distribution in both genders, (Figure 4).

### 3.4. Differences in the Percentage of Immune Cells According to Hypertension and Diabetes in COVID-19 Patients

**Figure 5:**
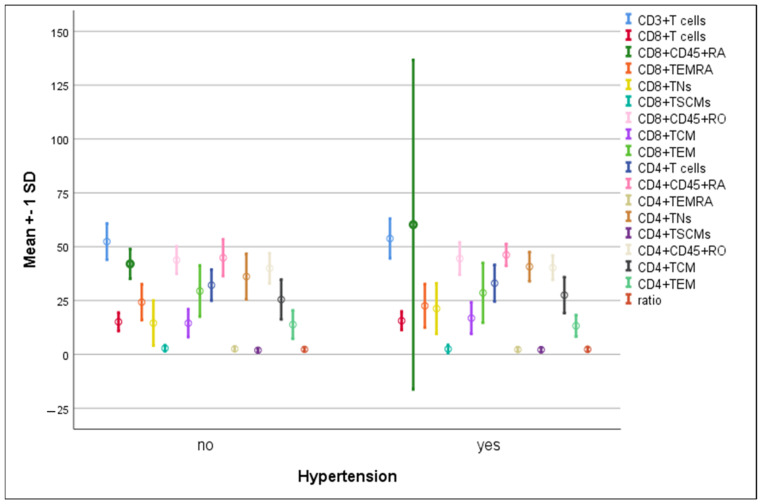
Differences in the percentage of immune cells according to hypertension in COVID-19 patients. Data are expressed as mean ± SD.

**Figure 6:**
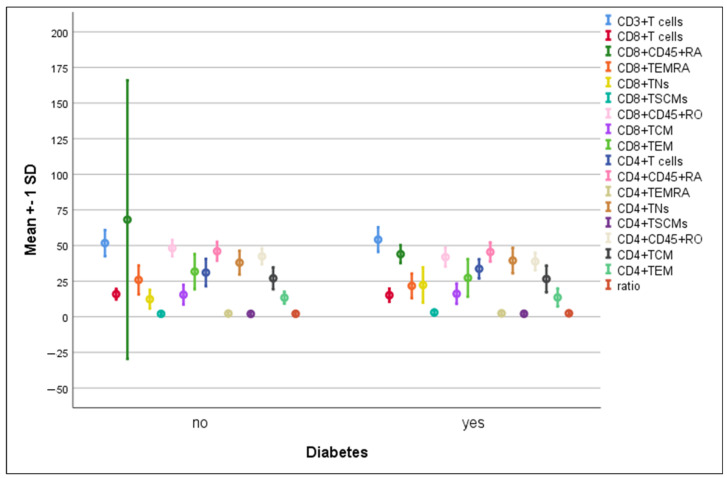
Differences in immune cells according to diabetes in COVID-19 patients. Data are expressed as mean ± SD.

There were no significant differences in the mean percentages of immune cells between hypertensive compared to non-hypertensive COVID-19-infected patients (Figure 5). The percentage of CD8^+^ TNs increased significantly in diabetic patients compared to non-diabetic ones (22.3 ± 12.5 vs. 12.3 ± 6.6, *p* = 0.004). On the contrary, a significant elevation in CD8^+^CD45RO^+^ cells was detected in non-diabetics compared to diabetics (48.2 ± 5.8 vs. 41.9 ± 6.7, *p* = 0.008) (Figure 6).

### 3.5. Relation of COVID-19 Severity to Immune Cells

**Figure 7:**
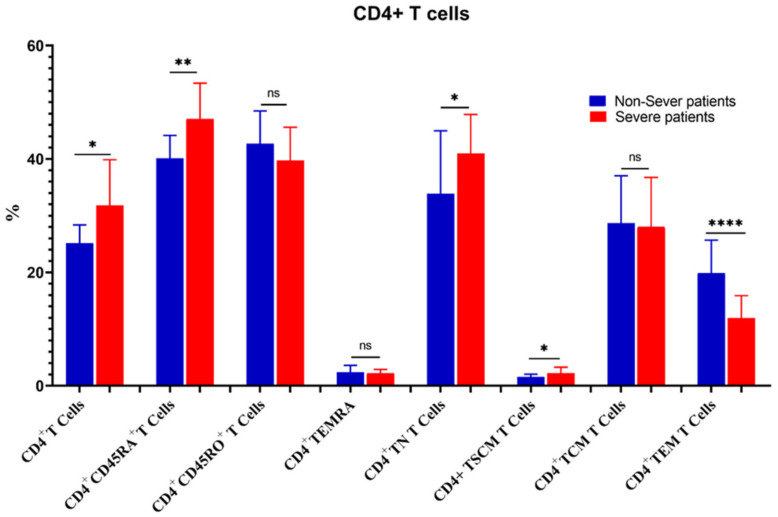
Comparison of the percentages of CD4^+^ T cells and their memory subsets in severe and non-severe COVID-19 patients. The ns indicated no significant differences among groups and the *p* values: * *p* < 0.05, ** *p* < 0.01, and **** *p* < 0.0001 indicated the significant correlation among different groups.

**Figure 8:**
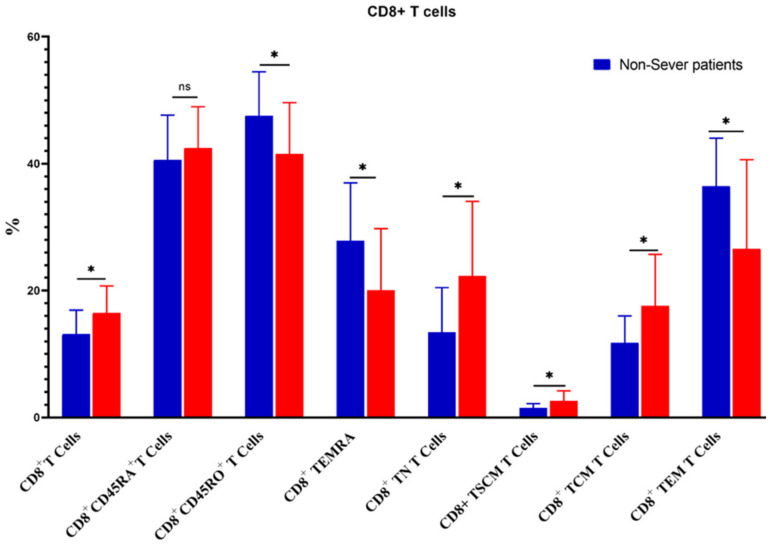
Comparison of the percentages of CD8^+^ T cells and their memory subsets in severe and non-severe COVID-19 patients. The ns indicated no significant differences among groups and the *p* values: * *p* < 0.05 specified the significant correlation among different groups.

The percentage of CD3^+^ T cells increased substantially in severe COVID-19 patients as compared to non-severe ones (55.01 ± 8.6 vs. 47.19 ± 7.2, *p* = 0.02). Similarly, CD4^+^ T cells, CD4^+^CD45RA^+^ CD4^+^ TN, CD4^+^ TSCM, CD8^+^ T cells, CD8^+^ TN, CD8^+^ TSCM, CD8^+^ TCM, and CD4^+^/CD8^+^ ratios were increased significantly in severe cases. On the contrary, the percentages of CD4^+^ TEM, CD8^+^CD45^+^RO, CD8^+^ TEMRA, and CD8^+^ TEM were significantly reduced in severe cases (Figure 7 and Figure 8).

## 4. Discussion

The manifestations of SARS-CoV-2 infection vary from one patient to another. Some patients have mild to moderate symptoms, while others have severe disease and may develop acute respiratory distress syndrome which necessitates hospitalization and mechanically-assisted ventilation [29]. Moreover, patients with COVID-19 exhibit increased risk of cerebrovascular insults including thrombosis, stroke, and pulmonary embolism, especially in critically ill patients such as diabetics, hypertensive, elderly (>65 years), and those with chronic obstructive pulmonary disease (COPD) [30,31,32].

Previous research findings have consistently demonstrated that SARS-CoV-2 infection-induced peripheral inflammation is correlated with the severity of the disease, with subsequent secondary immune mechanisms engaged in COVID-19 progression. This correlation highlights the critical role played by the immune system in influencing the outcome of SARS-CoV-2 infection and disease progression [33,34]. Understanding this interplaying immune responses and disease outcome is essential for advancing our knowledge of COVID-19 pathogenesis and developing effective strategies for managing and treating the disease.

Dysregulation in immune response and notable variations in immune phenotypes have emerged as prominent features in the context of COVID-19, particularly among individuals with severe manifestations of the disease [33]. In the present study, CD8+ T cells exhibited a significant decline in patients compared to healthy control, while CD4+ T cells were comparable between controls and patients. These findings are consistent with Mathew et al., [35] who reported deterioration in the percentage of CD8^+^ T cells in COVID-19 patients. Another investigation reported that there were no significant changes in CD4^+^ T cell subsets amongst COVID-19, convalescence, and healthy control people [36]. The current study also showed that SARS-CoV-2-specific CD4^+^ and CD8^+^ T cells were significantly elevated in the blood of severely infected patients compared to non-severe cases. We also found a significant elevation in CD8^+^ TEMRA level in patients compared to controls with a marked decline of CD4^+^ TEM, CD8^+^ TEM, and CD8^+^ TEMRA in severe cases. These findings are in agreement with Weiskopf et al., who provided evidence that SARS-CoV-2-specific CD4^+^ and CD8^+^ T cells appear in the blood of severely infected patients [37]. However, the same authors showed that SARS-CoV-2-specific CD4^+^ T cells typically had a central memory phenotype (CD4^+^ TCM), whereas CD8^+^ T cells generally had an effector phenotype (CD8^+^ TEM and CD8^+^ TEMRA) [37].

In this study, a positive correlation was elucidated between CD4^+^/CD8^+^ TSCMs and disease severity despite not being significantly different between the patient and control groups. Our findings are partially in line with De Biasi et al., who revealed that CD4^+^ and CD8^+^ TSCMs were comparable between COVID-19 patients and controls without being stratified by COVID-19 severity [38]. The link between CD4^+^/CD8^+^ TSCMs and the severity of COVID-19 can be explained based on the opposed correlation between TEM and TSCM cells, proposing that a compensating mechanism was rebuilt by TSCM cells’ differentiation, leading to a switch in these cell populations’ proportion to preserve immune system homeostasis [38]. Interestingly, our study showed that CD8^+^ TSCMs cells were activated in the blood of the recovered group compared to either the healthy control or COVID-19 patients. Similarly, a differentiated memory phenotype was recently defined for SARS-CoV-2–T cells, and has been well recognized by polyfunctionality and proliferative potential [39,40,41]. TSCMs’ ability to differentiate into different subsets of memory T cells might attribute to long-term immunity against SARS-CoV-2 in individuals who have recovered from COVID-19 [42]. Of note, the possible immune protective role of TSCM in SARS-CoV-2 needs further investigation in larger cohorts with longitudinal follow-up studies.

Following COVID-19 infection of the host cells, T cells can recognize viral peptide fragments and attack infected cells, but not pre-existing memory T cells as they have limited capacity to prevent infection. Later, these cells become rapidly activated to resolve viral infection and to enhance patients’ recovery. Moreover, a small proportion of CD4^+^ and CD8^+^ T cell populations will persist to form a long-lived memory cell pool that can re-activate following re-encountering COVID-19 infection [43]. Accordingly, many memory T cell subsets in our study did not increase in patients compared to healthy controls. However, induction of CD8^+^ TSCMs cells in the recovered group highlights the protective role of this special subset against SARS-CoV-2 and that their activation is positively correlated with the improved prognosis, as was postulated for HIV-1 infection [38]. More relevantly, SARS-CoV-1-specific T cells that may cross react with SARS-CoV-2 infection have been detected 17 years post-infection; this could partly explain why CD8^+^ TEM accumulated in healthy controls compared to patients. In addition, the pre-existing immune response in healthy controls might be due to the activated immune T cells by previous exposure to different types of corona viruses that also cross react with SARS-CoV-2 virus epitopes such as the common cold, causing coronaviruses [44,45].

It was postulated that chronic comorbidities such as diabetes are highly prevalent in COVID-19 patients and are correlated with an elevated risk of severe COVID-19 outcome and mortality [46]. Our results only detected significantly elevated CD8^+^ TNs in diabetic patients with COVID-19. Several works have reported heterogeneous T cell response in diabetic patients with COVID-19; some revealed significantly lower CD3^+^, CD4^+^, and CD8^+^ T cells [47,48], while others revealed decreased CD4^+^ T cells and increased CD8^+^ T cells in diabetics [49]. Furthermore, previous study reported significantly reduced TNs with increased CD4^+^ TEM and CD8^+^ TCM [50].

This study provides valuable insights into the intricate link between alterations in memory T cells and the severity of COVID-19. It sheds light on the potential role of these crucial immune compartments as indicators of disease severity, which can be invaluable for both understanding the pathogenesis of the virus and developing more precise diagnostic and therapeutic strategies. However, there are some limitations to consider. While this study aimed to examine the phenotypic alterations of memory T cells in response to COVID-19, the absence of molecular data is a drawback. Future research will be crucial in understanding the molecular effects of SARS-CoV-2 infection and COVID-19 on the development and function of memory T cells. Moreover, the study is limited by a small sample size and the lack of follow-up samples. To strengthen the statistical validity of the results, it is important to include a larger number of participants.

## 5. Conclusions

This study leveraged peripheral blood samples obtained from SARS-CoV-2 infected patients to comprehensively elucidate their immune responses in the context of the disease, focusing on in-depth profiling of T cells and their memory subsets. Alterations in subsets of T memory cells including CD4^+^ TSCM, CD8^+^ TSCM, and CD8^+^ TCM, CD4^+^ TEM, CD8^+^ TEM, and CD8^+^ TEMRA T cells have been linked to disease severity and associated comorbidities. A gender bias in the percentage of memory T cells in context of COVID-19 has been identified. This study sheds light on the dynamic changes that occur in the percentages of memory T cells during SARS-CoV-2 infection and their specific implications in patients with underlying comorbid conditions, thereby providing a more comprehensive understanding of the immune response dynamics in COVID-19 and potentially guiding the development of more targeted therapeutic interventions. The study represents a step forward in advancing our understanding of the pathology of COVID-19 and its immunological intricacies, paving the way for further investigations and advancements in the field. Future follow-up longitudinal studies involving the analysis of memory T cells subsets in blood samples of COVID-19 patients at different stages of the disease, encompassing active infection, recovery, and convalescence, hold the potential to gain deeper insights into the intricate interactions among immune cells especially during infection progression and in comorbid patients.

## Figures and Tables

**Table 1:** Demographics and comorbidities among COVID-19 patients.

Data	Control	Recovered COVID-19	COVID-19 Patients
Age (mean ± SE)	58.3 ± 3.5 y	60.2 ± 2 y	61.1 ± 1.5 y
Median (min–max)	57 (52–76)	59 (53–75)	60 (50–78)
Sex (m/f)	14/11	9/7	20/15
Hypertension	-	-	21 (60%)
Diabetes	-	-	22 (62.9%)
Severity of COVID-19	-	-	
Non-severe			8 (22.9%)
Severe			27 (77.1%)

Data are expressed as numbers, percentages, mean ± SE, median.

**Table 2:** Laboratory characteristics of COVID-19 patients.

Characteristic	Descriptive (Mean ± SE)
D-dimer (µ/mL)	3.7 ± 0.4
Ferritin (ng/mL)	643 ± 60.5
CRP (µg/mL)	106.0 ± 12.2
RBCs (million/mm^3^)	4.7 ± 0.2
Hemoglobin level	12.4 ± 0.4
Platelets (million/mm^3^)	259.1 ± 17.8
WBCs (million/mm^3^)	11.6 ± 0.8
Neutrophils (million/mm^3^)	10.4 ± 0.8
Lymphocytes (million/mm^3^)	0.95 ± 0.1
Monocytes (million/mm^3^)	0.63 ± 0.1
Eosinophils (million/mm^3^)	0.02 ± 0.01
Basophils (million/mm^3^)	0.025 ± 0.004

CRP: C-reactive protein, WBCs: white blood cells, RBCs: red blood cells.

## Data Availability

All data in this study are available and included in this published article.
